# Impacts of long-term land use and land cover change on land suitability potential in three sub-catchments of the Lake Tana Basin, Ethiopia

**DOI:** 10.1007/s10661-025-14806-9

**Published:** 2025-12-17

**Authors:** Rahel Seifu, Paul D. Wagner, Seifu A. Tilahun, Nicola Fohrer

**Affiliations:** 1 Department of Hydraulics and Water Resource Engineering, Gondar Institute of Technology, Gondar University, Gondar, Ethiopia; 2 Department of Hydrology and Water Resources Management, Kiel University, Kiel, Germany; 3 Applied Physical Geography, Environmental Hydrology and Resource Management, Institute of Geographical Sciences, Department of Earth Sciences, Freie Universität Berlin, 12249 Berlin, Germany; 4 Civil and Water Resources Engineering, Bahir Dar Institute of Technology, Bahir Dar University, Bahir Dar, Ethiopia

**Keywords:** Land use and land cover change, Lake Tana Basin, Land suitability, MCA-AHP, GIS & RS

## Abstract

Population growth and agricultural expansion cause major changes in land use and land cover (LULC) in Ethiopia. Cultivated lands are mostly expanding without land suitability evaluation. Consequently, crop yields are not increasing as expected. This is particularly the case in the highland catchments draining toward Lake Tana, where severe consequences such as deforestation and the degradation of soil and land can be observed. In this study, the impacts of long-term LULC dynamics on the land suitability potential for selected major crops in three sub-catchments of Lake Tana, Ethiopia (Gilgelabay, Gumara and Ribb), were evaluated. Time series of Landsat images from three periods (1988, 1998, and 2017) were classified. Land suitability was analyzed via a multi criteria approach based on spatial input data such as elevation, soil, and slope maps. The overall accuracy for all LULC classifications was good to very good (89.7% to 91.6%). Five major LULC classes were distinguished: agriculture, forest, shrub/bushland, grassland, and water. In all three catchments, the results revealed that agricultural land was the dominant land cover that expanded at the expense of the other land cover types to 80%-90% in all catchments in 2017. The rate of change in agricultural land in the Gilgelabay catchment (4041.3 ha/yr) was greater than that in the Gumara (1374.5 ha/yr) and Ribb (1362.3 ha/yr) catchments. This is possibly due to the availability of other LULC classes. The natural vegetation of Gilgelabay, Gumara, and Ribb has decreased by 16.0%, 10.5%, and 1.1%, respectively, over the past three decades. However, the present LULC change trends are unsustainable, and any remaining natural vegetation should be maintained. The results from the land suitability analysis revealed that the land suitability for teff, corn, and rice is likely to change with climate change in the future. To ensure sustainable land use management, modifying land use on the basis of land suitability should be preferred over traditional practices to improve crop production. This can be achieved in close collaboration with all stakeholders, including local communities, the government, and NGOs.

## Introduction

Land use and land cover (LULC) change is a significant component of global environmental change and is one of the major factors affecting sustainable development (Lambin et al., 2001; Turner et al., 2007). Except for those areas that are inaccessible, most of the Earth’s surface has already changed from a natural state to anthropogenic land uses (Turner et al., 1993; Williams et al., 2020). Moreover, 38% of agricultural areas on Earth can be considered degraded (Backer et al., 2009; Ngugi, 2020). The largest proportion of degraded land can be found in Africa (UNEP, 2016). At least 485 million (65%) people in Africa are affected by land degradation, corresponding to 46% of the continent’s land area and 75–80% of the continent’s arable land (AGNES, 2020). Ethiopia is one of the countries in Sub-Saharan Africa that is most susceptible to land degradation processes because of its heavy reliance on agriculture.

In Ethiopia, forest cover was approximately 40% in the late nineteenth century (Amogne, 2014; Sisay & Gitima, 2020). The clearing of land for agricultural use and deforestation for fuel gradually reduced forest areas from 40% in 1900 to 16% in 1954, 8% in 1961, 4% in 1975, 3.2% in 1980, and less than 3% in 2014 (Amogne, 2014; Sisay & Gitima, 2020). The northern parts of the highlands are almost devoid of trees (Lombardi & Deer Lodge, 2012). Agriculture in Ethiopia is the foundation of the country’s economy, accounting for half of the gross domestic product (GDP), including 90% of foreign exchange and 85% of total employment (Gebreyesus, 2016; Chanie et al., 2018; FAO, 2019 and Neglo et al., 2021). An efficient use of land is necessary to meet the demand for food. A prerequisite for such an efficient use of land for agriculture is its evaluation with respect to growing certain crops on the basis of biophysical factors and the region’s socio-economic situation (Dang & Kawasaki, 2017; FAO, 1976). In Ethiopia, the majority of the population is rural (> 80%) and works in the agricultural sector, which provides 95% of the country’s demand for food (Esa & Assen, 2017). Moreover, Ethiopia has high agricultural potential because of its wide productive land, sufficient rainfall, and labor force (Admassie & Abebaw, 2021; Agidew, 2015). However, despite this potential, Ethiopia has experienced several food crises during recent decades because it is dependent mainly on rain-fed agriculture (Kisaka et al., 2015). By considering biophysical and socio-economic factors, land can be assessed to measure the quality of land for a particular use (Bagherzadeh et al., 2011). This evaluation of land is important for efficient and sustainable land use, taking into account the limitations of natural resources (Qureshi et al., 2018). It is helpful for land users, land use planners, and agricultural organizations to overcome limitations and increase productivity (Rabia, 2012).

According to the Food and Agricultural Organization (FAO, 1976, 1983), climate, soil requirements, and land terrain information should be considered for each crop to assess land suitability (Hamere and Teshome, 2018). The FAO (1976) and FAO (1983) also recommended an approach for the evaluation of land suitability for crops by rating areas from highly suitable (S1) to not suitable (N) areas on the basis of elevation, rainfall, temperature, slope, and soil texture. Several parts of the world are severely affected by climate change (Mudzonga, 2011; Okonya et al., 2013; Kibret & Robai, 2015 and Fahad & Wang, 2020). These changes affect land suitability, particularly the production of rain-fed crops. Climate change affects future agricultural productivity, but its impact varies by region, time, and socio-economic development (Kibret & Robai, 2015; Schlenker & Lobell, 2010). In many parts of the highlands of Ethiopia, agriculture has gradually expanded from gently sloping land into the steeper slopes of neighboring mountains without protective measures against erosion and degradation. Population growth is a driver of these LULC changes. According to the Central Statistics Agency (2017), the projected population growth rate of Ethiopia increased by 2.67% per year from 1955 to 2020. Projected estimates show that the country’s population will double in 40 years (Montgomery, 2008). Moreover, in the Ribb catchment, population growth has been found to have led to a decline in forest and bushland (Nurelegn & Amare, 2014; Getachew & Manjunatha, 2022); in forest, bushland, and grassland in the Gumara catchment (Chakilu & Moges, 2017); and in forest and shrubland in the Gilgelabay catchment (Rientjes et al., 2011) of the Lake Tana basin. Similarly, Tadesse and Hailu, (2024) and Zegeye (2024) reported that population growth is the main cause of land degradation, poverty, and food insecurity in the northern Ethiopian highlands.

Previous studies have generally indicated that forest cover is decreasing at the national level, biodiversity has been lost, and soil and environment have been degraded across the country (Dibaba et al., 2020; Sisay & Gitima, 2020). This finding has implications for the stream flow of the Nile River and the downstream countries, Sudan and Egypt. In recent centuries, Ethiopian highlands have degraded due to continuous cultivation, causing more overland flow and less base flow (Girma et al., 2023; Nzuza et al., 2021). The greater amount of surface runoff from land degradation, like deforestation, leads to a faster hydrological response; thus, this water will remain usable only if additional storage facilities are built. However, retaining rainwater has become increasingly difficult because of the loss of forest cover, which results in sedimentation and a reduced volume in reservoirs (Tilahun et al., 2013). Kidane et al. (2019) reported that LULC change had a significant effect on erosion, sediment yield, and water quantity in the Legedad Reservoir catchment in Ethiopia (Mengstu, 2016). Moreover, the intensification of agricultural land has continuously decreased water quality.

Crop land suitability has been assessed in different regions of Ethiopia. In the Upper Blue Nile Basin, Yalew et al. (2016) identified regions with high suitability for agriculture and those with relatively high susceptibility to land degradation and soil erosion. Girmay et al. (2018) reported that land suitability can be enhanced by soil and water conservation measures, improving soil fertility and agro-economic practices in the northeastern part of the highlands. In the Gelda catchment, northwest highlands of Ethiopia, Esa and Assen (2017) examined the suitability status of plots of land to choose a range of alternative land utilization types with the same suitability level. According to Yohannes and Soromessa (2018), the lower half of the Andit-Tid catchment in the highlands of Ethiopia is characterized primarily by marginally suitable land with severe limiting characteristics such as soil depth, texture, slope, and temperature, as well as erosion risk. Moreover, competition for land is high because of the demand for food and space as a result of population pressure, resulting in the degradation of resources (Hamere and Teshome, 2018; Yohannes et al., 2021; Admasu et al., 2023). Therefore, increasing crop yield by growing crops in the locations most suited to their growth is essential (Cassman et al., 2010). However, the topography of the Blue Nile Basin in Ethiopia, where the study area is located, is very rugged, dissected, and mountainous, which aggravates the problems of soil erosion and nutrient depletion and has a direct effect on the water fluxes in the catchment. To adapt to possible future effects of LULC changes on water resources, it is important to understand the hydrologic impacts of historic LULC changes. Additionally, the expansion of agriculture, growth of urban areas, and extraction of timber and other natural resources are likely to accelerate over the coming decades to satisfy the growing demands for land, wood, and food. Population growth and livestock density in the Lake Tana basin have resulted in forest clearing and overgrazing. In addition, more mountainous areas and steeper slopes are cultivated, in many cases without protective measures against erosion and degradation. Consequently, there is a discrepancy between agricultural land use and crop requirements. Crop production systems are dominated by smallholder farming under rain-fed agriculture with little mechanization. The productivity of cereals is still very low (CSA, 2018), and the fact that the land’s suitability for crops is not considered is one of the primary reasons for the low yield. Suitable areas are used for a range of alternative land utilization types due to, e.g., population growth, a high percentage of croplands, or less awareness of farmers of land suitability. Previous studies have so far not focused on the effects of long-term changes in LULC and their impacts on land suitability in the Lake Tana Basin in Ethiopia.

The aim of this study was to examine the impacts of long-term LULC changes on the land suitability potential of selected agricultural sub-catchments of the Lake Tana Basin. More specifically, we aimed to determine LULC changes between 1988 and 2017 by dividing the period into ten-year intervals, comparing and identifying the leading causes of LULC changes, assessing major crop suitability and finally identifying whether the change in agricultural land was based on suitability. In addition, we assess future rain-fed agricultural land suitability maps for major crops on the basis of measured and future projected climate data (RCP4.5 and RCP8.5), topography, and soil properties. This information will help local governments, policymakers, land-use planners, and agricultural stakeholders formulate and implement effective and appropriate response strategies to minimize the undesirable effects of future LULC changes or modifications. Understanding the impacts of LULC is critical for resource-based studies and for developing effective response plans for sustainable agricultural productivity, food security and environmental stability in the country and the study area.

## Materials and methods

### Study area

Lake Tana is the largest lake in Ethiopia. It is situated between 10.95° and 12.78°N latitude and 36.89° and 38.25°E longitude and is a part of the upper Blue Nile Basin. The lake covers an area of 3,063 km^2^, has a drainage area of approximately 15,096 km^2^ (Ligdi et al., 2010), and has more than 40 tributary rivers (Dejen et al., 2017). Lake Tana receives more than 93% of the stream flow from the Gilgelabay, Ribb, Gumara, and Megetch catchments, which are found in the regional state of Amhara (Setegn, 2010). The three sub-catchments of Gilgelabay, Ribb, and Gumara were selected as study areas. They are the most densely populated areas with a population density of more than 250 people/km^2^, with a total of approximately 4.0 million inhabitants in the basin (World Bank, 2009). Agriculture is the basis for the livelihoods of more than 80% of the population living in the study area. In addition, the economic activity of the population depends on agriculture and livestock production. Hilly areas and steeper slopes are more often farmed, frequently without protection from erosion and damage. The most common soil types are Haplic Luvisols, Eutric Leptosols, Eutric Fluvisols, Chromic Luvisols, and Eutric Alisols (Tigabu et al., 2019).

The Gilgelabay River has a catchment area of approximately 3,559 km^2^ and is the largest tributary of Lake Tana, contributing 44.2% of the inflow (Table 1). It originates from a highland spring in the city of Gish-Abay in northeastern Ethiopia (Wale et al., 2008). Geographically, Gilgelabay is located south of Lake Tana between 10° 56′ 53″−11° 21′ 58″N and 36° 49′ 29″−37° 23′ 34″E.

**Table 1:** Characteristics of the Gilgelabay, Gumara, and Ribb catchments(Tigabu et al., 2020)

Catchment	Catchment area upstream of gauge (km^2^)	Mean annualtemperature1980-2014 (°C)	Mean annual rainfall sum1980-2014 (mm)	Mean elevation (m)	Mean flow 1980–2014 (m^3^/s)
Gilgelabay	3559	19	1550	2270	55
Gumara	1229	16	1464	2260	34
Ribb	1584	16	1475	2374	14.4

**Fig. 1:**
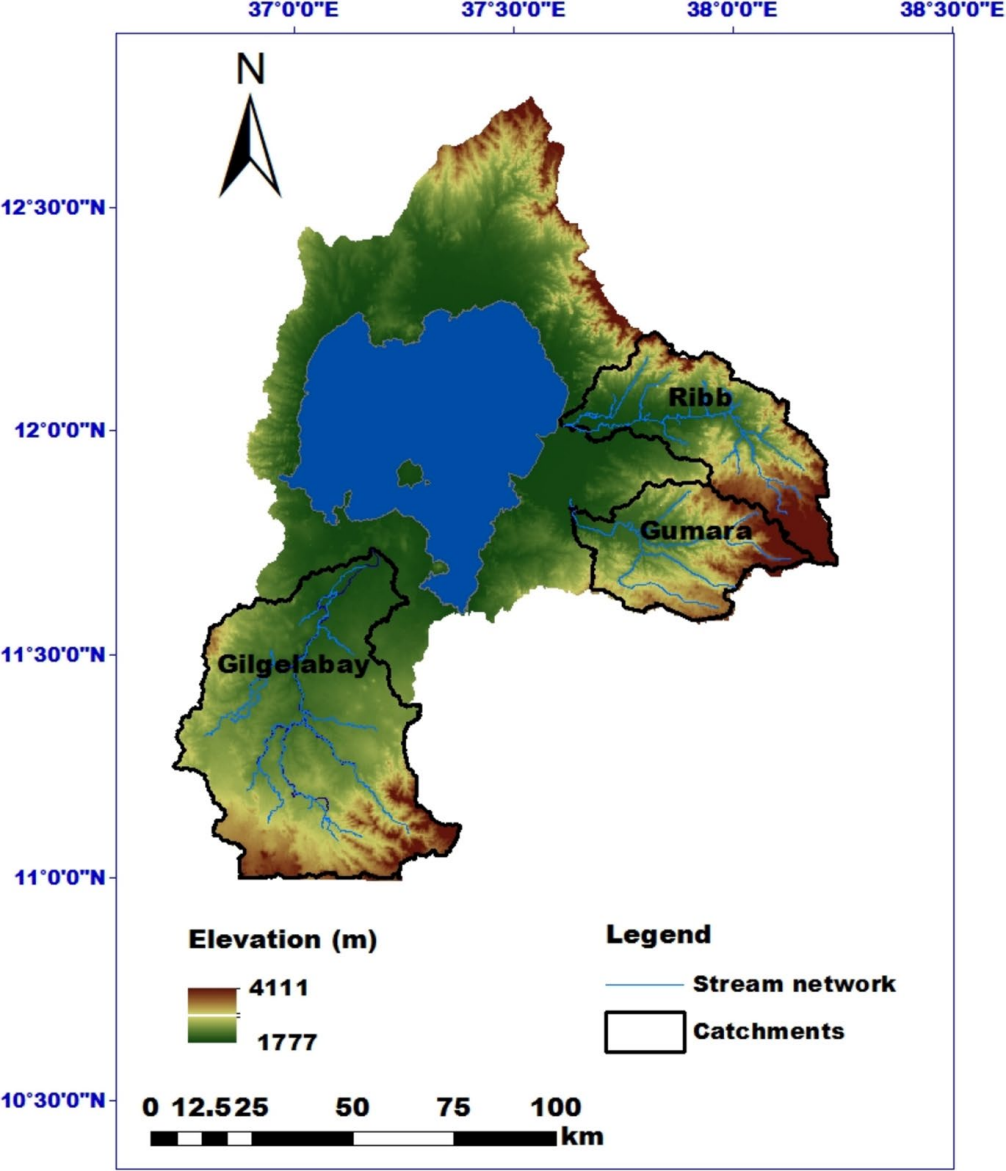
Location map of the Gilgelabay, Ribb, and Gumara catchments in the Lake Tana basin

The Gumara catchment is located east of Lake Tana between 11° 35’−11° 55’ N and 37°40’- 38° 10’ E (Fig. 1) and covers an area of 1,229.8 km^2^ (Table 1). It has mountainous and dissected terrain. Steep slopes characterize the upper stream, and gentle slopes form the downstream parts of the catchment. The area is drained by numerous smaller streams, including Gumara, the largest stream in the catchment that drains into Lake Tana (Wubie et al., 2016).

The Ribb River is another tributary to Lake Tana in the east and originates from Mount Guna (Adem et al., 2016). The catchment has an area of 1,584 km^2^ and is located between 11°42′20″—12°11′11″ N and 37°42′43″—38°14′20″E (Fig. 1).

The main rainfall season in the study area is from June to September and accounts for 70–90% of the annual rainfall (Abdo et al., 2009).

### Land use and land cover change analysis

#### Satellite data

Cloud-free multi-spectral satellite data for three points in time were obtained. Landsat satellite images from 1988, 1998, and 2017 were used (Table 2). Two satellite images were required to cover the study areas: 169/52 (path/row) for the catchments of Gumara and Ribb and 170/52 for Gilgelabay. All images were taken during the dry season because the phenological difference between wet-season vegetation and dry-season vegetation in tropical regions can result in pseudo-changes in LULC. This reduces the confusion of pasture and small-scale rainy season agriculture.

**Table 2:** Satellite data specifications

Year	Satellite	Sensor	Path	Row	No. of spectral bands	Spatial resolution	Acquisition date	Source
1988	Landsat 5	TM	169/170	52	7	30 m × 30 m	14/13 March 1988	USGS
1998	Landsat 5	TM	169/170	52	7	30 m × 30 m	18 March/10 April 1998	USGS
2017	Landsat 8	OLI, TIRS	169/170	52	11	30 m × 30 m	7/13 March 2017	USGS

### Land use and land cover classification

The selected cloud-free, geometrically rectified and projected satellite images were classified using a supervised classification approach with a maximum likelihood classifier. In this classification, each pixel is assigned to the class that has the highest probability for this particular class (Sisodia et al., 2014). To this end, ground truth data, which were collected during a field survey in 2017, are needed. Since mapped ground truth data for 1988 and 1998 were not available, ground truth data were interpreted from satellite images using different band combinations (Tucker et al., 2004) and high-resolution Google Earth images (Wagner et al., 2013; Wilken et al., 2017). The number of samples of ground truth points was based on the area of the catchments: 260 points were used for Gilgelabay, and 250 points were used for Gumara and Ribb. For 1988 and 1998, the accuracy assessment was based on ground truth from image interpretation and from Google Earth, whereas for 2017, additional field survey data were used. The pixels in the training locations were normally distributed and represented the study area well.

The following five major LULC types have been mapped in the study area: agricultural land, forest, grassland, shrubland, and water (Table 3).

**Table 3:** Description of LULC class types

LULC class	Description
Agriculture	Areas with standing crops, tree crops, and croplands where the crops were harvested (FAO, 1997)
Forest	Land covered by natural and planted trees, including bamboo, that reach a maturity height of more than 2 m, have a canopy cover of more than 20% and cover more than 0.5 ha with a minimum width of 20 m (MEFCC, 2018)
Grassland	Ground covered by vegetation dominated by grasses (FAO, 1997)
Shrubland	Shrub vegetation at the fringes of forest cover and areas dominated by scattered trees less than 5 m in height (FAO, 1997)
Water	Areas with surface water in the form of ponds, lakes, streams, rivers, and reservoirs (FAO, 1997)

#### Accuracy assessment

An accuracy assessment is necessary to quantify the reliability of the produced LULC classification. From the confusion matrix, user and producer accuracies can be calculated for each LULC class (Story & Congalton, 1986). The overall accuracy is defined as the total number of correctly classified pixels divided by the sum of the ground truth pixels. The F1 score is the harmonic mean of precision and recall, ranging from 0 to 1, and is computed for each LULC class separately (Tharwat, 2021).

1 $$\mathrm{F}1\text{ score}=2*\mathrm{UA}*\mathrm{PA}/ (\mathrm{UA}+\mathrm{PA})$$

Where PA = producer accuracy and UA = user accuracy (Ouma et al., 2023).

#### Change detection

The patterns and changes in major LULC classes between 1988, 1998, and 2017 were analyzed across all three catchments using the derived LULC classifications. The percentage areas for each LULC class as well as the changes in spatial patterns were compared. Moreover, the rate of LULC change was calculated for each class as follows:

2 $$\mathbf{r}=({\mathbf{Q}}_{2}-{\mathbf{Q}}_{1})/\mathbf{t}$$

Where r is the rate of change, Q1 is the initial year LULC class area in ha, Q2 is the recent year LULC class area in ha, and *t* is the time difference between initial and recent LULC classes in years (Alemayehu et al., 2019). LULC conversions were assessed with the help of a conversion matrix and expressed as percentages of total change.

### Land suitability analysis

#### Climate and soil data

**Fig. 2:**
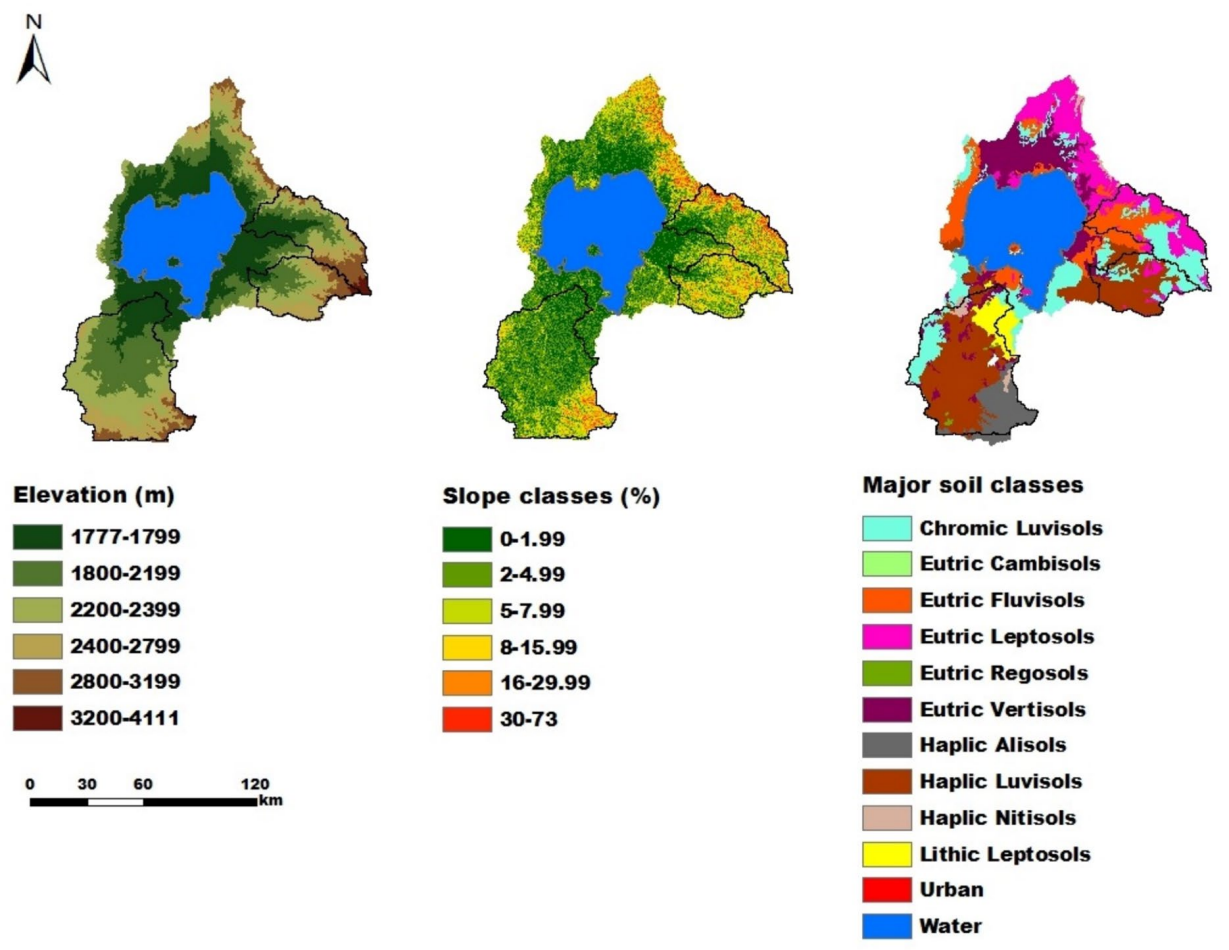
Elevation (left), slope (middle) and soil (right) maps of the Lake Tana basin

**Fig. 3:**
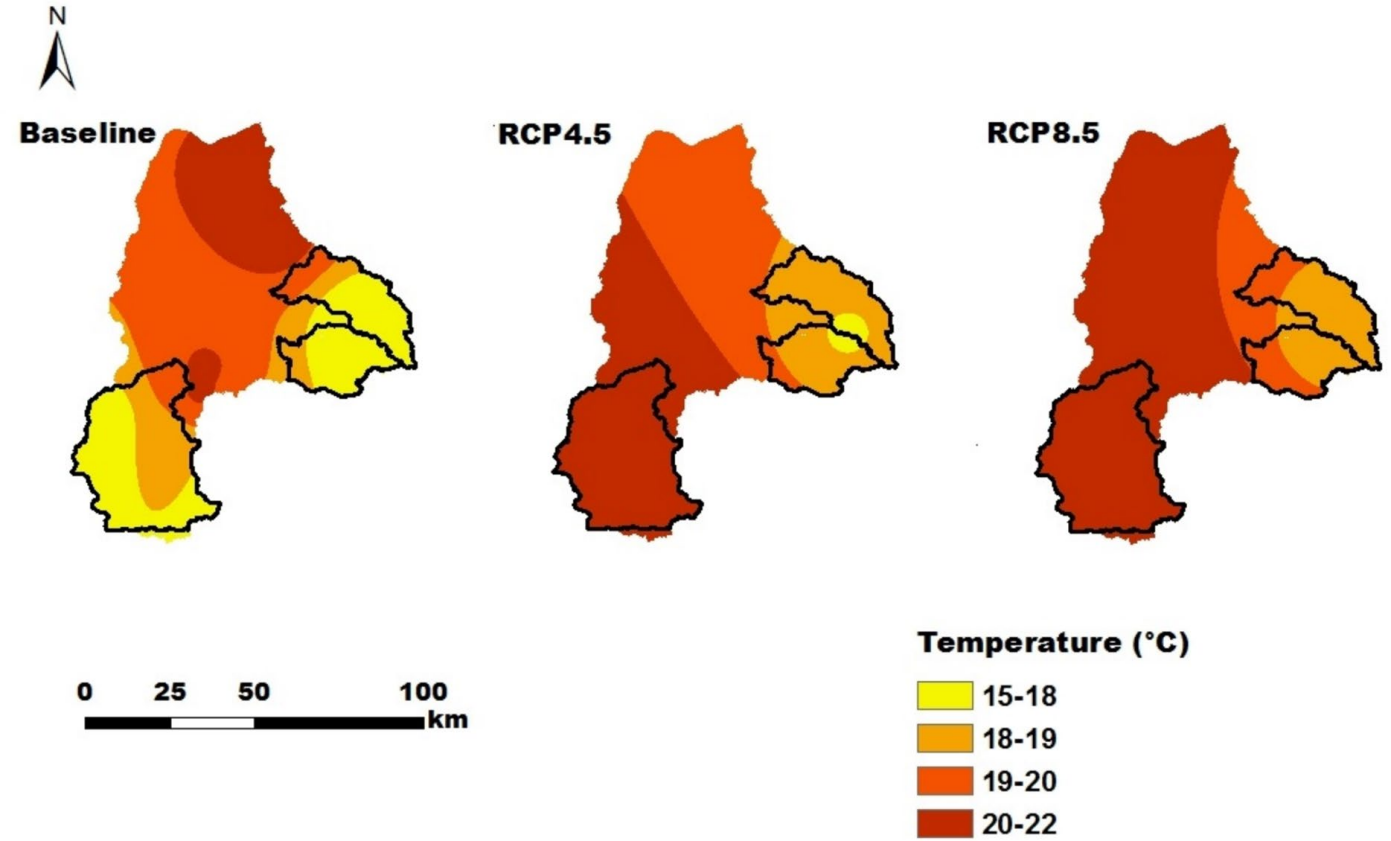
Average temperature distributions in the Lake Tana Basin under the Baseline, RCP4.5 and RCP8.5 scenarios in °C

**Fig. 4:**
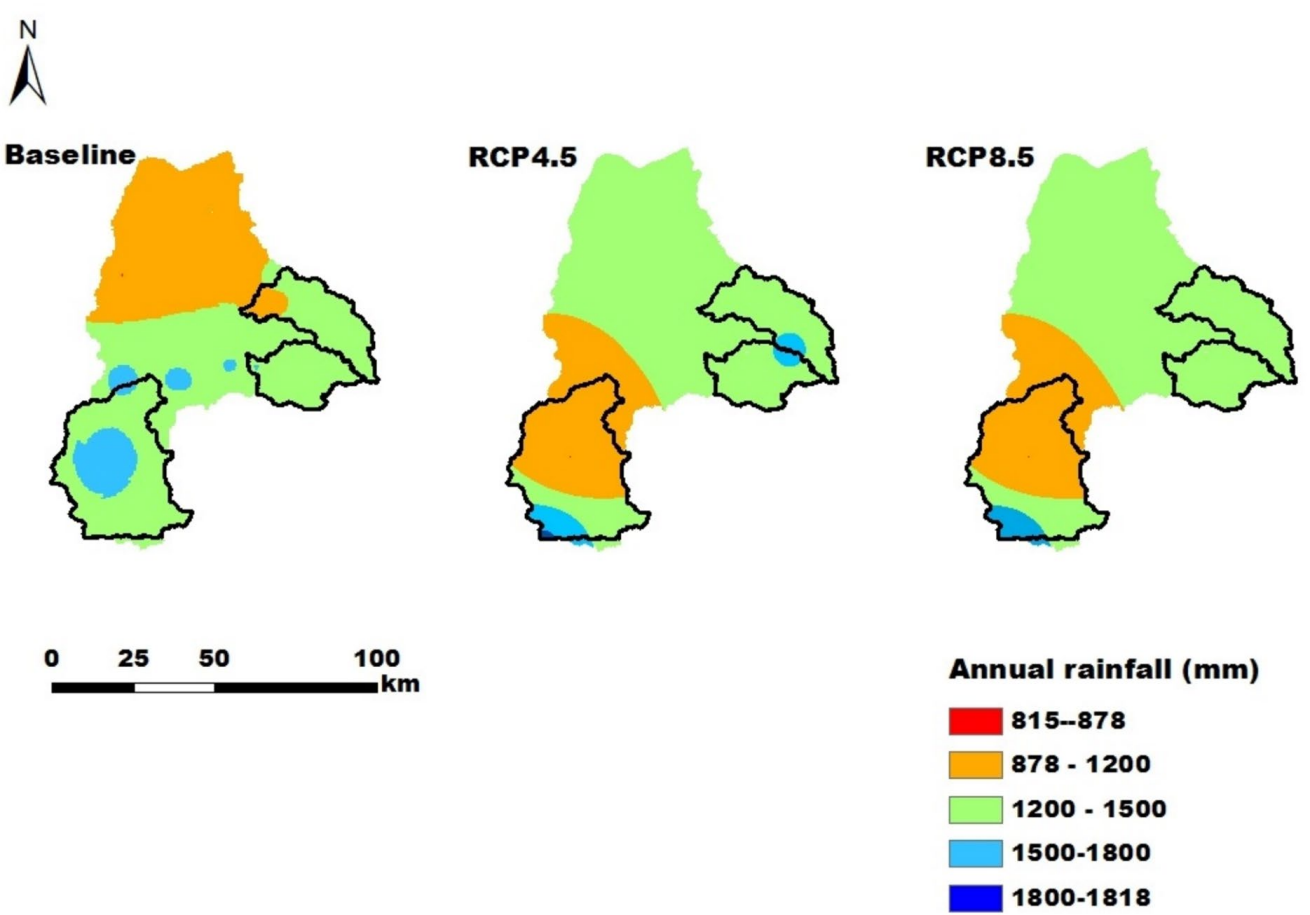
Annual rainfall distributions of Lake Tana Basin for the Baseline, RCP4.5 and RCP8.5 scenarios in mm

A soil map (Fig. 2) from the Amhara Design & Supervision Works Enterprise (ADSWE, 2017) and thirty years of temperature and rainfall data (1985–2015) from the Ethiopian National Meteorology Agency (ENMA) were obtained. A detailed analysis of historic climate data in the study area is presented in Tigabu et al. (2020). For future projections, the midterm (2031–2060) projected RCP4.5 and RCP8.5 temperature and rainfall data (Figs. 3 & 4) from regional climate models (RCMs) were used. RCM outputs provide better spatial and temporal resolutions than GCM (Global Circulation Model) outputs do. However, projected temperature and rainfall are still biased (Berg et al., 2012; Tigabu et al., 2021). Hence, bias correction (BC) methods were used. Table 4 lists the chosen GCM and regional climate model (RCM) combinations, along with their full names, institutions, and BC methods (Tigabu et al., 2021). Consequently, we calculate the ensemble mean to derive more robust scenario results. In addition, the slopes and elevations of the study areas (Fig. 2), which are essential for land suitability analysis, were derived from a Digital Elevation Model (DEM) with a spatial resolution of 30 m that is based on the Shuttle Radar Topography Mission (SRTM) (USGS, 2016; Tigabu et al., 2019).

**Table 4:** Summary of selected GCMs and RCMs, the applied bias correction (BC) method, and the calculated root mean square error (RMSE) values (Tigabu et al., 2021)

GCM/RCM	Institution name	Expanded name of RCM	Bias correction (BC) method	RMSE before BC (mm/day)	RMSE (mm/day)	Suitable GCM–RCM
CCCma/RCA4	Canadian Centre for Climate Modeling and Analysis	Regional-scale model	pLS (linear scaling)	2.32	0.35	2
MIROC/RCA4	RCA4		pDM (distribution or quantile mapping)	6.4	0.16	2
MPIM/RCA4	Max Planck Institute for Meteorology	Regional-scale model	pDMLIS (distribution mapping and local intensity scaling)	3.34	0.5	2
NCC/RCA4	National Climate Center of China		pLS (linear scaling)	2.32	0.2	2
Total						8

#### Matching crops with requirements

Suitability evaluation is a process of matching crops with their specific land use requirements and is performed employing the maximum limitation method for the specified land utilization types/crops (FAO, 1983; Hamere and Teshome, 2018; Yohannes & Soromessa, 2018). This method defines the land suitability classes by taking the most limiting factors into account (details are provided in Table 1 in the appendix). To derive suitability classes, selected parameters are matched with the requirements (elevation, slope, rainfall, temperature, and soil) of selected crops and are weighted for each factor on the basis of the analytic hierarchy process (AHP). In this instance, rain-fed crops were selected for crop suitability evaluation, including teff and corn for the Gilgelabay catchment and teff, rice, and corn for the Gumara and Ribb catchments. The crops were selected on the basis of their dominance in the respective catchments.

#### Calculation of weights for criteria maps

To analyze complex decisions, the analytic hierarchy process (AHP) is a recommended technique that divides alternatives into pairwise alternatives of two at a time (Liu, 2022; Saaty, 2008, 2013). According to Wale et al. (2012), weighting by pairwise comparison is more appropriate than equal weighting and ranking. A relative importance measure is used in the pairwise comparison (Tables 5 and 6). Weighting the factors is an essential step that requires expert knowledge. Hence, experts were requested to rank the factors used in this study (i.e., rainfall, temperature, elevation, soil, and slope) and to allocate weights according to how important the factors are for crop growth.

**Table 5:** Pairwise comparison matrix for multi-criteria decision layers

Factors	Elevation	Slope	Rainfall	Temperature	Soil	Criteria weight
Elevation	1.00	2.00	0.33	0.50	3.00	0.17
Slope	0.50	1.00	0.50	0.33	2.00	0.12
Rainfall	3.00	2.00	1.00	3.00	3.00	0.38
Temperature	2.00	3.00	0.33	1.00	4.00	0.26
Soil	0.33	0.50	0.33	0.25	1.00	0.07

**Table 6:** Fundamental scale for the pairwise comparison matrix (Alemayehu, 2020; Khan & Khan, 2022)

Relative importance	Definition	Description
1	Equally important	Two criteria enrich equally to the objective
3	Slightly important	Judgments and experience slightly favor one criterion over another
5	Fundamentally important	Judgments and experience strongly favor one over the other
7	Really important	One is strongly favored and its dominance established in practice
9	Absolutely important	Evidence favoring one criterion over another is of the highest probable order of affirmation
2,4,6,8	Adjacent	Used when intermediate importance is needed

Analytic hierarchy processes use the pairwise comparison matrix to calculate comparative weights for each factor and produce a consistency ratio (CR) that serves as a measure of the logical inconsistency of expert/user judgment (Yalew et al., 2016). Hence, consistency relationships are important for determining possible events and measuring the logical inconsistencies of decision makers and judgments (Cengiz & Akbulak, 2009; Chen et al., 2010a, 2010b).

The consistency relationship (CR) is used to measure the efficiency of the criteria of the AHP employing Eq. 1 (Yalew et al., 2016).

3 $$\mathrm{CR}=\mathrm{CI}/\mathrm{RI}$$

Where CR is the consistency ratio, CI is the consistency index, and RI is the random index.

The value of the CR should be less than 0.1 (Yalew et al., 2016). If the value of CR > 0.1, then some pairwise values need to be reconsidered and the process should be repeated until the value of CR is < 0.1.

4 $${CI}={\lambda max}-{n}/({n}-1)$$

Where λmax represents the highest eigenvector of the computed matrix and n represents the order of the matrix. The random inconsistency indices are derived from Pramanik (2016, Table 7).

**Table 7:** Random inconsistency indices (RIs) (Pramanik, 2016)

n	1	2	3	4	5	6	7	8	9	10
RI	0	0	0.58	0.90	1.12	1.24	1.32	1.41	1.46	1.49

Maximum eigenvalue (λmax) = 5.3, number of factors (n) = 5 and RI = 1.12.

CI = 0.08.

CR = 0.07.

#### Standardization of criteria and the rating of suitability classes

The data were measured on different units and different scales. Hence, it is essential to standardize the factors prior to combination and ensure that they are transformed, and each factor map has a positive correlation with suitability (Kenzong et al., 2022; Yohannes & Soromessa, 2018). During reclassification, factor ratings were also assigned for values that indicate how well each factor is satisfied by particular conditions of the corresponding land quality. All the criteria maps have been reclassified into classes. The land suitability map is classified into four classes: highly suitable, moderately suitable, marginally suitable and not suitable (FAO, 1976 & 1983) (Table 8, and Table A10).

**Table 8:** Physical land suitability classification and descriptions used to standardize the data into these suitability classes (FAO, 1976; Alemayehu, 2020). The parameter ranges for each class and the analyzed crops are provided in the appendix (Table A11)

Code	Class	Characteristics
S1	Highly suitable	Land has no limitation for a given use. No need for a high level of input
S2	Moderately suitable	Land has minor limitations that could reduce productivity or benefit. Adaptive inputs are required to reach the same yield as that of class S1
S3	Marginally suitable	Land has moderate limitations for specific use, in which the amount of surplus input is only marginally justified
N	Not suitable	Land with severe limitations for land use is under consideration

**Fig. 5:**
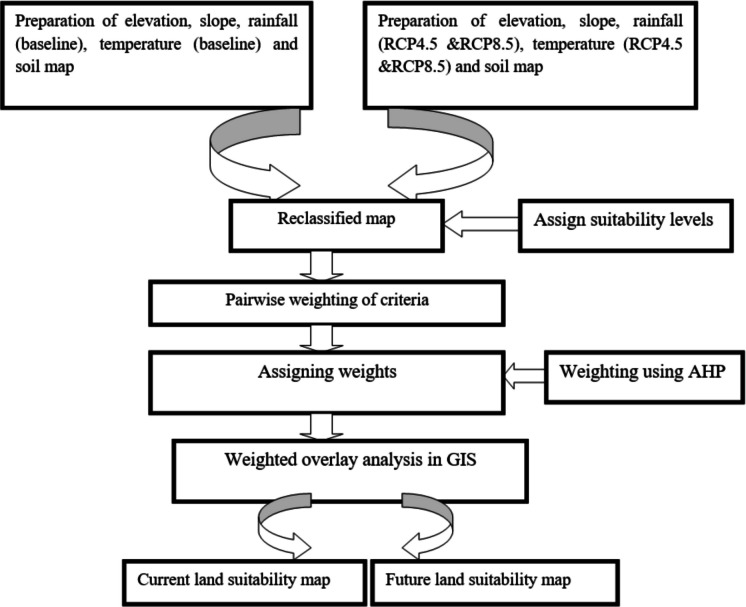
Flow chart for the analysis of the land suitability of crops

Land suitability analysis was carried out to assess the land suitability for current and projected climate data (RCP4.5 and RCP8.5) with the same elevation, soil, and slope data of Gilgelabay, Gumara, and Ribb for teff, rice, and corn crops (Fig. 5).

#### Detection of the suitability of agricultural land change

It is critical to detect changes in agricultural land expansion to comprehend past, present, and future tendencies in change. To detect the suitability of agricultural land change, corn is selected because of its wide range of suitability criteria compared with teff and rice (Appendix Table A10). In addition, corn accounts for a larger proportion of the land suitability area than teff and rice in all three catchments (Appendix Table A11). The change map of agricultural land was compared with the suitability map to assess whether the expansion of agricultural land between 1988 and 2017 was based on suitability.

## Results

### Land use/land cover assessment

**Fig. 6:**
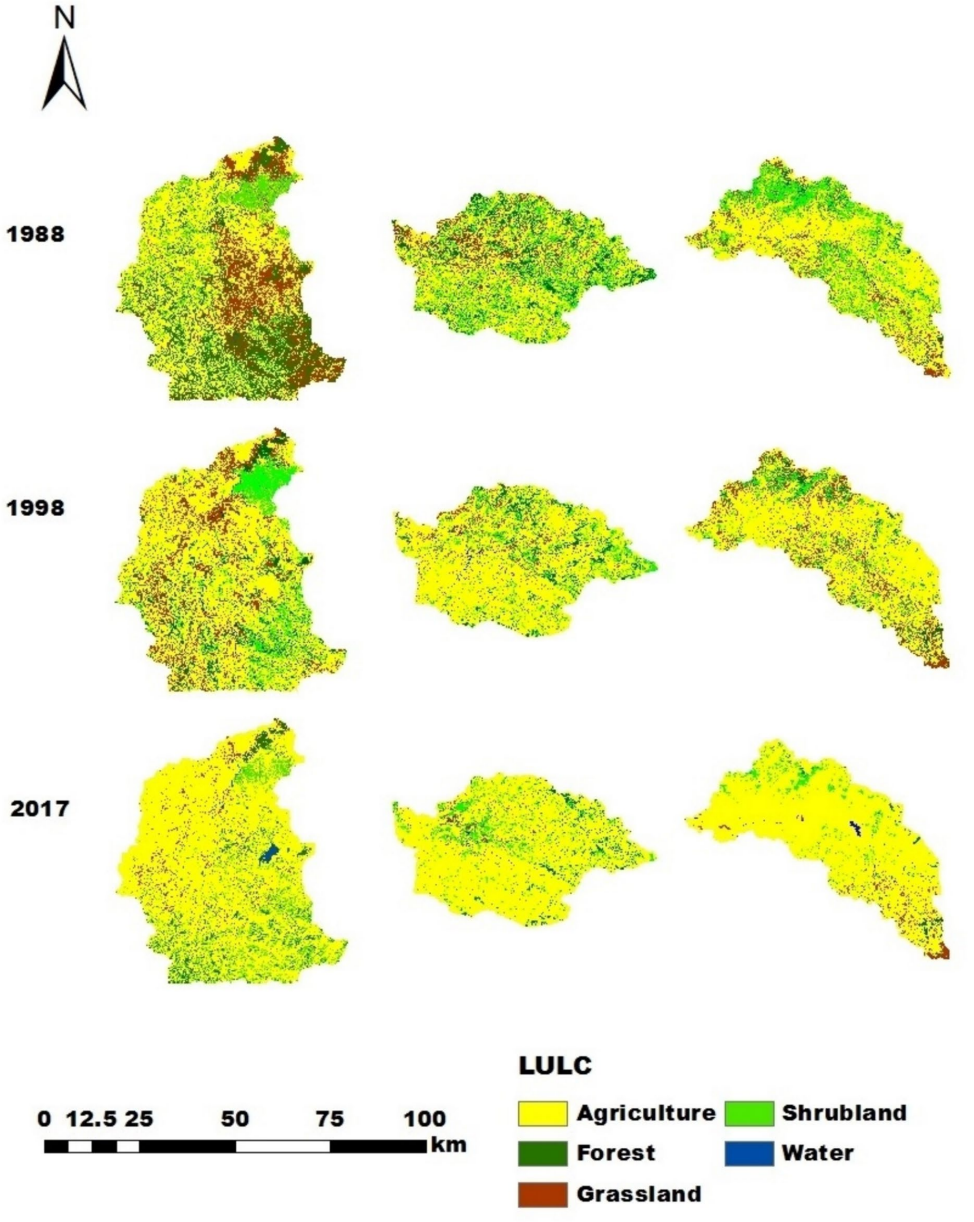
LULC maps of the Gilgelabay, Gumara, and Ribb catchments in 1988, 1998, and 2017

**Fig. 7:**
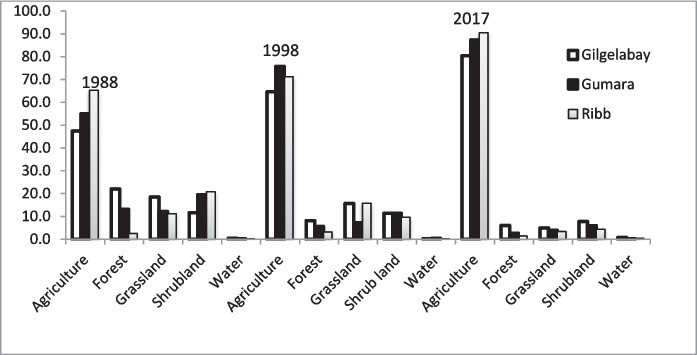
Percentage of LULC classes in the Gilgelabay, Gumara, and Ribb catchments for the years 1988, 1998, and 2017

In 1988, agricultural land represented the largest portion of the Gilgelabay catchment (47.5%), followed by forest (22.0%) (Figs. 6 and 7). The percentages of grassland, shrubland, and water were 18.5%, 11.6%, and 0.4%, respectively. In 1998 and 2017 in Gilgelabay, the percentage of land allocated for agriculture increased to 64.6% and 80.4%, respectively. Consequently, in 1998, the percentages of forest, grassland, and shrubland decreased to 8.1%, 15.6%, and 11.5%, respectively, and in 2017, they decreased to 6.0%, 5.0%, and 7.7%, respectively. The water area increased to 0.76% of the catchment area in 2017, which may have been related to the construction of the Koga Reservoir in 2011.

Similarly, the 1988 LULC classification of the Gumara catchment revealed that agricultural land (54.9%) constituted the largest percentage of the catchment area. Here, shrubland (19.5%) was the second dominant land cover, whereas forest (13.2%), grassland (12.2%), and water (0.3%) occupied smaller portions. In the Gumara catchment, the percentage of land allocated for agriculture increased to 75.7% (1998) and 87.3% (2017), and the percentages of forest, grassland, and shrubland decreased to 5.6%, 7.2%, and 11.3% in 1998 and to 2.6%, 4.0%, and 6.0% in 2017, respectively. During the entire analysis period (1988–2017), the water area decreased by 0.17%.

Analysis of the 1988 classification of the Ribb catchment also revealed that agricultural land (65.3%) constituted the largest percentage of the catchment area. Like in the Gumara catchment, shrubland (20.8%) is the second dominant land cover type in the Ribb catchment. The percentages of forest, grassland, and water were 2.5%, 11.2%, and 0.2%, respectively. In 1998, the classification revealed that the percentages of land allocated for agriculture, forest, grassland, and water increased to 71.2%, 3.2%, 15.8%, and 0.3%, respectively, whereas shrubland decreased to 9.7%. The increase in forest area in 1998 might have been due to the conservation measures taken due to the policy of “forest conservation, development and utilization” enacted in 1994 to reduce land degradation and increase soil fertility to increase agricultural production. In 2017, a further increase in land allocated for agriculture (90.5%) and water (0.43%) was observed, but the percentages of forest, grassland, and shrubland decreased to 1.4%, 3.3%, and 4.4%, respectively. The increase in water might be related to the construction of the Ribb Dam in 2016.

The image classification results were validated using ground truth data and a confusion matrix (Appendix Table, 1, 2, 3, 4, 5, 6, 7, 8, 9). The agreement in the catchments of Gilgelabay, Gumara, and Ribb is excellent for all time steps (Table 9). Additionally, the class-specific accuracies indicate good to very good accuracy for all classes at all time steps (Table 10)

**Table 9:** Overall accuracyfor the 1988,1998, and 2017 LULCmaps

	Overall accuracy 1988	Overall accuracy 1998	Overall accuracy 2017
Gilgelabay	89.7%	90%	90.4%
Gumara	90.4%	91.6%	90.8%
Ribb	90.4%	91.2%	90.4%

**Table 10:** Minimum and maximum user and producer accuracies for the 1988, 1998, and 2017 LULC maps

	Gilgelabbay(1988–2017)		Gumara (1988–2017)		Ribb (1988–2017)	
	User accuracy	Producer accuracy	User accuracy	Producer accuracy	User accuracy	Producer accuracy
Agriculture	88.8–91.7	91.3–92.8	90.4–92.9	92.9–94.4	91.4–94.0	92.0–93.0
Forest	88.6–91.5	88.6- 93.1	86.5–93.6	85.3–86.5	82.6–90.5	83.3–88.4
Grassland	83.9–87.5	75.0- 79.5	86.7–93.3	83.9–84.9	86.7–90.7	84.8–89.7
shrubland	87.5–88.0	84.6- 90.0	83.6–93.3	90.3–92.12	88.2–90.4	88.2–90.0
Water	95.5–100	91.3–100	100	100	100	100

### LULC change analysis

**Fig. 8:**
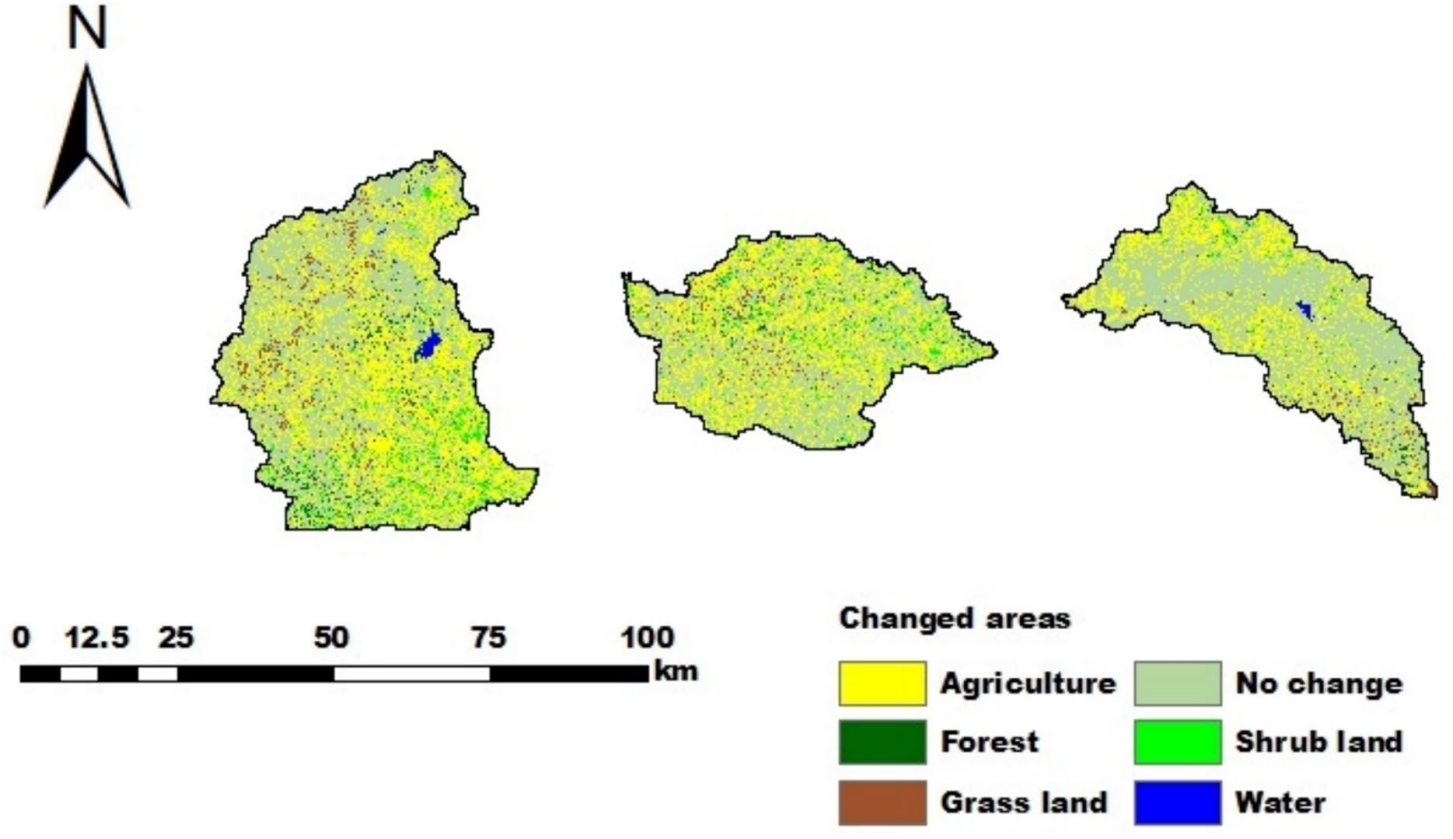
Changed LULC depicted with the LULC of 2017 and the areas that were not changed compared with the LULC of 1988 for the Gilgelabay, Gumara, and Ribb catchments

**Fig. 9:**
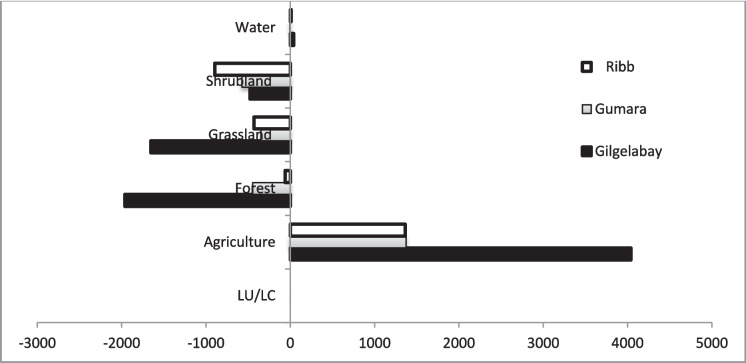
Rate of change in LULC classes between 1988 and 2017 in ha/year for the Gilgelabay, Gumara, and Ribb catchments

Approximately 49.3%, 46.7%, and 33.0% of the Gilgelabay, Gumara, and Ribb catchments changed between 1988 and 2017, respectively (Fig. 8). Population growth is a major driver of these changes, resulting in a growing demand for food, fuel wood, and construction materials. The expansion of agriculture is due mainly to the conversion of forest, grassland, and shrubland into agriculture. However, this is also slightly different for the catchments (Table 11). Forest areas were particularly affected (Fig. 8, 9), as forest clearing is also a source of income, particularly if no other income is available. These changes affect the water balance (Tigabu et al., 2019) and potentially impact Lake Tana and downstream water users.

**Table 11:** Land use and land cover conversion matrices for Gilgelabay, Gumara, and Ribb. Changes are expressed in percent of the land use and land cover classes in 2017

Gilgelabay		Gumara		Ribb	
LULC category	Agriculture	LULC category	Agriculture	LULC category	Agriculture
Agriculture	53.9	Agriculture	57.0	Agriculture	69.0
Forest	16.7	Forest	10.4	Forest	1.6
Grassland	17.7	Grassland	12.7	Grassland	10.2
Water	0.3	Water	0.2	Water	0.1
Shrubland	11.4	Shrubland	19.6	Shrubland	19.1
LULC category	Forest	LULC category	Forest	LULC category	Forest
Agriculture	16.8	Agriculture	23.5	Agriculture	42.0
Forest	65.1	Forest	59.7	Forest	38.5
Grassland	15.4	Grassland	2.2	Grassland	7.6
Water	0.2	Water	0.2	Water	0.0
Shrubland	2.6	Shrubland	14.4	Shrubland	12.0
LULC category	Grassland	LULC category	Grassland	LULC category	Grassland
Agriculture	33.4	Agriculture	51.4	Agriculture	37.1
Forest	33.4	Forest	12.9	Forest	1.2
Grassland	27.8	Grassland	16.9	Grassland	42.9
Water	0.1	Water	0.3	Water	0.1
Shrubland	5.3	Shrubland	18.5	Shrubland	18.6
LULC category	Water	LULC category	Water	LULC category	Water
Agriculture	6.5	Agriculture	66.6	Agriculture	65.4
Forest	21.9	Forest	1.4	Forest	0.2
Grassland	48.4	Grassland	12.6	Grassland	3.4
Water	19.6	Water	15.2	Water	24.2
Shrubland	3.7	Shrubland	4.1	Shrubland	6.8
LULC category	Shrubland	LULC category	Shrubland	LULC category	Shrubland
Agriculture	17.9	Agriculture	39.2	Agriculture	18.1
Forest	36.1	Forest	33.6	Forest	10.7
Grassland	20.1	Grassland	5.2	Grassland	8.9
Water	0.2	Water	0.4	Water	0.0
Shrubland	25.7	Shrubland	21.6	Shrubland	62.2

When the three catchments are compared, the rate of change in agriculture in the Gilgelabay catchment is greater than that in the Gumara and Rib catchments (Fig. 9). This may be because the availability of land, which can be converted into agricultural land, was greater in the Gilgelabay catchment than in the Gumara and Ribb catchments (i.e., the availability of other land in 1988 was 52.5% in Gilgelabay, 45.1% in Gumara, and 34.7% in Ribb). Consequently, the rate of change for decreasing forest and grazing land is also the greatest in Gilgelabay.

### Land suitability

#### Weight factors

The analytic hierarchy process (AHP) was used to calculate weights for factor maps by dividing them pairwise. The weights were assigned on the basis of the specific requirements of crops and on the knowledge of farmers and experts (Table 6). Climatic (temperature and rainfall) and topographic (elevation) factors were the dominant criteria suggested by the experts because of the influence of climate on rain fed agricultural crops.

#### Land suitability analysis for major crops

**Fig. 10:**
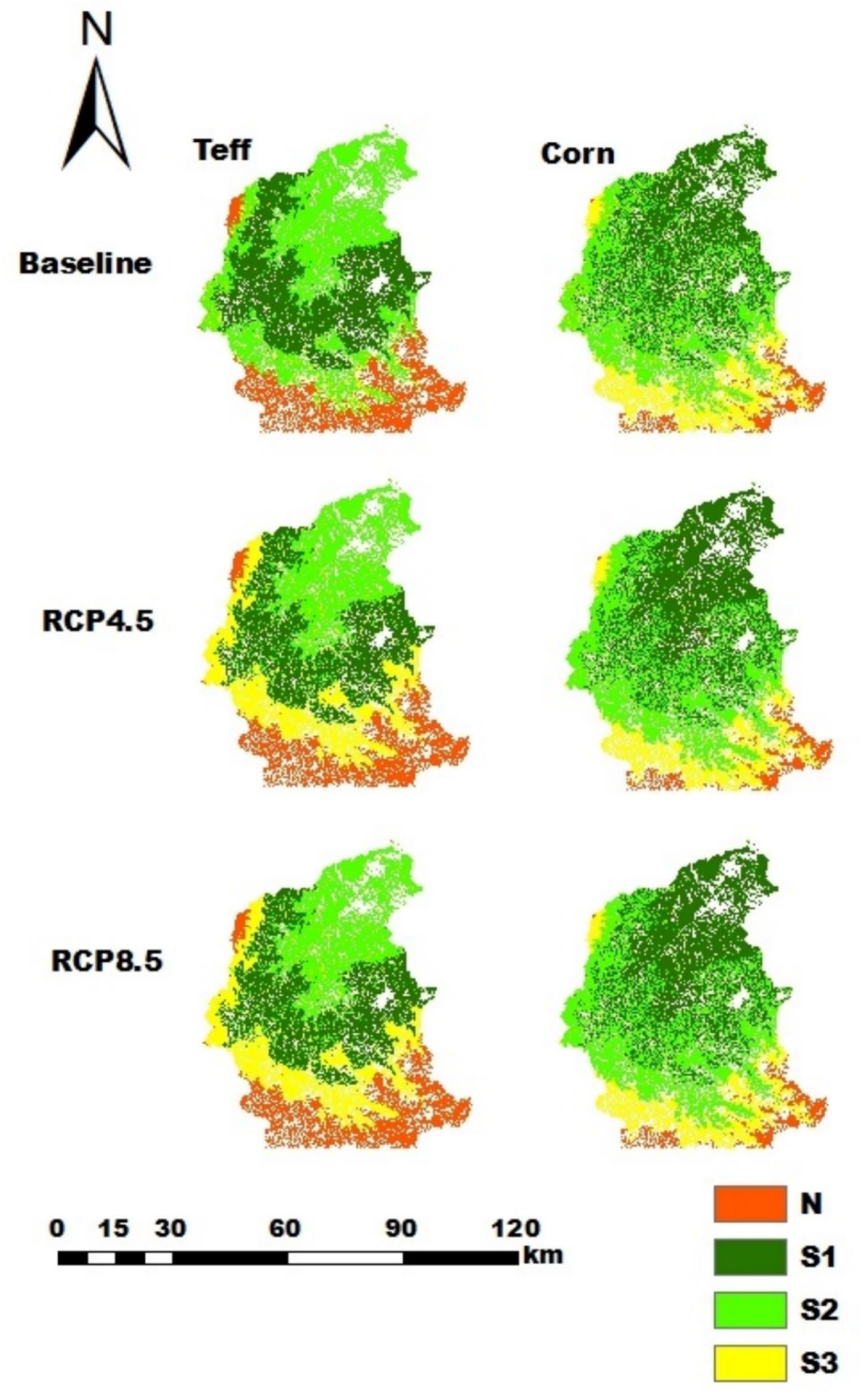
Land suitability maps for teff and corn in the Gilgelabay catchment for the current climate and the RCP4.5 and RCP8.5 future scenarios

In the Gilgelabay catchment, teff and corn are the most widely harvested crops. The current suitability map of Gilgelabay indicates that the percentages of highly suitable land for teff and corn were 33.1% (95,003 ha) and 33.4% (96,057 ha), respectively. Under the RCP4.5 and RCP8.5 projected climate data for the years 2031–2060, the percentages of highly suitable land for teff and corn were 26.3% (75,552 ha) and 38.0% (109,260 ha), respectively. These changes are related to changes in rainfall and temperature. The remaining moderately suitable, marginally suitable, and not suitable classes (Table 8, Table A11) indicate that the analyzed factors (elevation, soil texture, temperature, rainfall, and slope) limit the growth of the considered crops (Fig. 10).

**Fig. 11:**
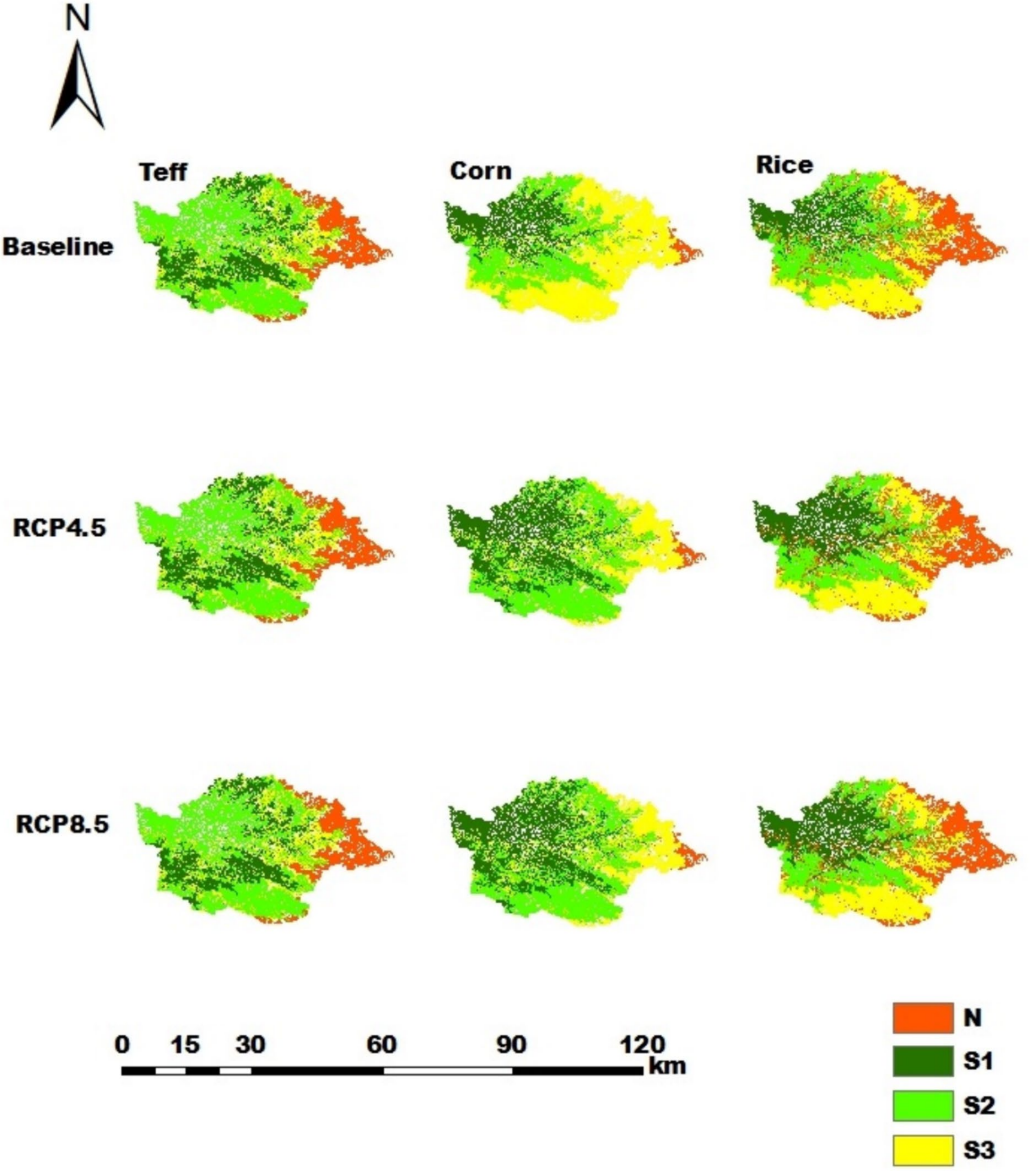
Land suitability maps for teff, corn, and rice in the Gumara catchment for the current climate and the RCP4.5 and RCP8.5 future scenarios

In the Gumara catchment, teff, rice and corn are the dominant crops (Fig. 11). The percentages of highly suitable land for teff, corn, and rice were 21.4% (23,327 ha), 20.6% (22,439 ha), and 17% (18,548 ha), respectively. By using RCP4.5 and RCP8.5 projected climate data for the years 2031- 2060, the percentage of highly suitable land for teff remains the same as that in the baseline. However, rice and corn increased to 22.9% (24,961 ha) and 34.8% (37,990 ha) of the catchment area, respectively.

Furthermore, for the baseline, moderately suitable (49.2%, 32.1% and 28.6%), marginally suitable (14.3%, 25.9% and 48.6%) and not suitable land (15.1%, 25.0% and 2.3%) were assessed for teff, rice and corn, respectively.

**Fig. 12:**
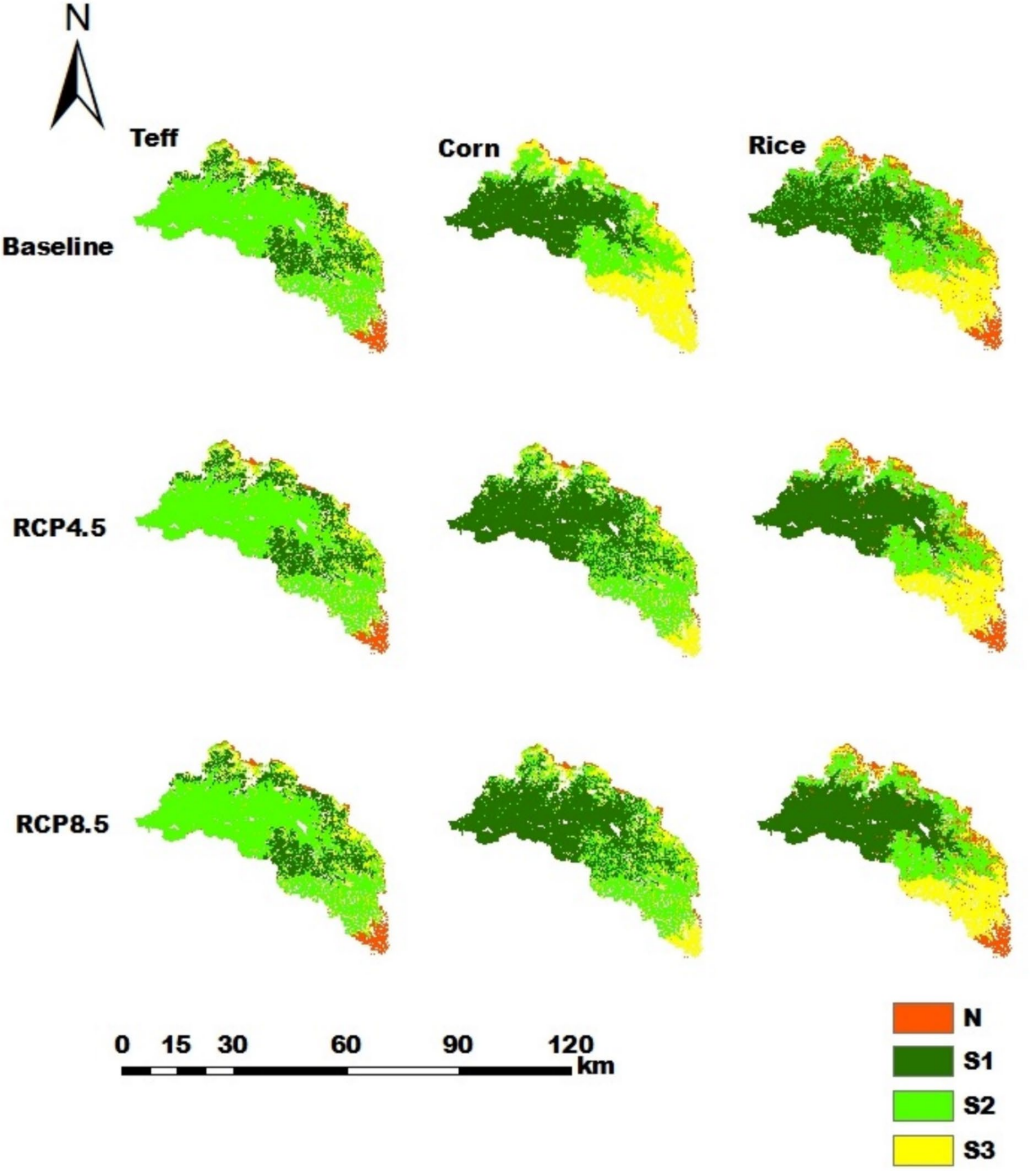
Land suitability maps for teff, corn, and rice in the Ribb catchment for the current climate and the RCP4.5 and RCP8.5 future scenarios

Figure 12 shows that in the Ribb catchment, teff, rice, and corn are the dominant crops, and the proportions of highly suitable land were approximately 20.2% (28,894 ha), 36.6% (52,407 ha), and 42.1% (60,391 ha), respectively. In addition, the projected climate data from 2031–2060 revealed that the proportion of highly suitable land remained the same for teff but increased for rice and corn to 54.8% (78,610 ha) and 43.9% (62,859 ha), respectively.

#### Detection of the suitability of agricultural land change

The suitability of agricultural land change was assessed by intersecting the corn suitability maps of the baseline (2017) and the change maps of the studied catchments (Table 12). Among the land used for agricultural expansion, marginally suitable land had the highest percentage, followed by moderately suitable land in all the studied catchments (40.8%, 46.6%, and 51.6% in Gilgelabay, Gumara, and Ribb, respectively). Hence, most of the observed past land expansion occurred in marginally suitable agricultural areas. This might be related to population growth and a high percentage of croplands or to farmers being less aware of the suitability status of land.

**Table 12:** Detection of the suitability of agricultural land expansion

Catchments	Suitability Class	Percentage
Gilgelabay	N	1.1%
	S1	22.3%
	S2	35.8%
	S3	40.8%
	**Total**	**100%**
Gumara	N	1.1%
	S1	14. 2%
	S2	38.1%
	S3	46.6%
	**Total**	**100%**
Ribb	N	0.9%
	S1	4.7%
	S2	42.7%
	S3	51.7%
	**Total**	**100%**

## Discussion

In the Ethiopian highlands, population growth has led to LULC changes (Hussein, 2023; Meshesha et al., 2016). Our analysis revealed pronounced changes between 1988 and 2017 in all three catchments. Owing to population growth, agricultural land throughout all three catchments increased at the expense of other land cover types. The decrease in forest cover is related to the expansion of agricultural lands and the use of wood as construction material, fuel, and an additional source of income.

Figures 6 and 7 indicate that the agricultural land in the Gilgelabay, Gumara, and Ribb catchments has increased to 80.38%, 87.34%, and 90.45%, respectively. However, the forest cover of Gilgelabay, Gumara, and Ribb has decreased by 6.03%, 2.63%, and 1.42%, respectively, over three decades (1988–2017) because it is influenced by population growth. The significant increase in agricultural land agrees with findings of Rientjes et al. (2011) for the upper Gilgelabay catchment between 1973 and 2001. Wubie et al. (2016) reported that cultivated land increased from 75.2% to 96.5% in the Gumara catchment and that forest, shrub/bush, grazing land, and wetland areas decreased from 13.1%, 8.4%, 2.7%, and 0.7% to 1.9%, 0.7%, 0.7%, and 0.2%, respectively, within 48 years (1957–2005). Nurelegn and Amare (2014) reported that within the period of 1973–2011 in the Ribb catchment, the cultivated area increased from 51.7% to 70.4%, and the forest, shrubland, and water areas decreased from 11.7%, 29.3%, and 0.5% to 5.0%, 14.0%, and 0.2%, respectively. Minale (2013) also found that between 1973 and 2008, the percentage of cultivated land increased from 26.1% to 41.2% over a 35-year period. Agriculture rapidly replaced forest, grassland and shrubland. One of the main factors influencing LULC changes is the expansion of agriculture, which is linked to population growth (Abuhay et al., 2023; Berihun et al., 2019; Hussein, 2023; Rientjes et al., 2011; Tewabe & Fentahun, 2020). This is perhaps due to the absence of coordination between the land distribution policy and the family planning policy. Moreover, the degradation of vegetation, such as forest, shrub/bushland, and grassland, can be observed. Abebe and Minale (2017) also reported that LULC changes were associated with poor land use policy implementation, low technical and financial capacity, little to no incentives, and frequent restructuring of key institutions at the national and regional levels. Additionally, land use planning can support community development by assisting the state in land resource management techniques, promoting appropriate land use, and protecting land resources to predict sustainable development and food security. Agriculture is the main economic activity in the study areas; therefore, the observed changes are directly related to agricultural development. The expansion of agricultural land at the expense of other LULC classes found in this study is well supported by the literature.

According to Eshetu (2014), recent land tenure in Ethiopia can be classified into three time periods. Before 1975, land tenure was based on a feudal system where land was concentrated in the hands of landlords and the church (Chala, 2016). Tenure rights were highly insecure. Owing to the overthrow of the feudal regime in 1974 and the Marxist-oriented government of the Derg, ownership of all rural land was given to the state for the distribution of use rights to cultivators through local peasant associations. The further transfer of land rights through sales, leases, and inheritance was very restricted. The government that took power in 1991 following the fall of the Derg, however, followed a free-market philosophy that has made little change to farmers’ land rights, which are still considered inadequate. Farmers mentioned that land management throughout catchments is poor because of land tenure policy. They noted that if land became individually owned, inhabitants would be more committed to sustainable land management (Nurelegn & Amare, 2014). Hence, changes in land tenure policy may support sustainable land management. Decreasing soil productivity over several years is another factor for the expansion of agricultural lands (Gomes et al., 2019). Farmers expand their plots simply by clearing forests and farming steep slopes to achieve a higher yield instead of using fertilizer because of its high cost. Nurelegn& Amare (2014) also reported that farmers do not have enough land, they do not use chemical fertilizers because of high financial costs, and no technique to manage soil loss is applied. Afforestation programs have been implemented in different years in the past (Takele et al., 2022). An afforestation program was carried out in Ethiopia between 2011 and 2015. In 2019, the government initiated the Green Legacy and organized an afforestation program that broke world records by planting 350 million trees daily and 4 billion trees in total. However, a mechanism should be established to protect planted trees. It can be recommended to counteract deforestation and land degradation by changing the land tenure policy, protecting planted trees, reducing the cost of fertilizer, and making inhabitants aware of sustainable land management practices.

For the RCP4.5 and RCP8.5 climate scenarios, the proportions of suitability classes of major crops in all catchments have the same values. Hence, the impacts of the considered midterm future climate predictions of RCP4.5 and RCP8.5 were not sensitive to land suitability. This is due to the fact that differences in the temperature and rainfall values under RCP4.5 and RCP8.5 were small in the midterm future prediction, resulting in the same suitability classes. In the Gilgelabay catchment, the baseline proportion of highly suitable land for corn was approximately 33.4%, but when the projected climate data for RCP4.5 and RCP8.5 were used, the proportion of highly suitable land increased to 38.0%. This is related to an increase in the average temperature from 15–20 °C in the baseline period to 20.6–21 °C and 21.2–22 °C for RCP4.5 and RCP8.5, respectively. The temperature ranges in the future scenarios strongly overlap with the highly suitable range for corn (17.5–22.5 °C, Table A11).The proportions of highly suitable areas for rice in the catchments of Gumara and Ribb were greater for both RCP4.5 and RCP8.5 than for the baseline, i.e., from 17.0% and 36.6% to 22.9% and 43.9%, respectively. This might be associated with an increase in the annual mean rainfall, i.e., greater than 1400 mm, which is highly suitable for rice (Table A11). According to Alemayehu et al. (2020) and Fekadu and Negese (2020), the amount of rainfall in the area is ideal for enabling the growth and development of these crops. This shows and suggests how climate change affects crop production in the future and, consequently, smallholder farmers’ livelihoods.

The area of highly suitable land for teff is greater in Gilgelabay than in Gumara and Ribb under all climate conditions. This can be explained by the availability of land and other climatic and topographical factors. In Ribb, the highly suitable land for rice was greater than that in the Gumara catchment. This might be explained by a gentler slope and a larger percentage of lowlands because lower elevations are associated with higher temperatures, resulting in a more suitable environment for rice. On the basis of these findings, climatic factors (temperature and rainfall) and topographic factors (elevation) are the dominant factors influencing the suitability of teff, corn, and rice. Areas with high elevations (1800–2200 m) and lower temperatures (15–20 °C) are most suitable for teff, and areas with low-to-medium elevations (1500–2200 m) and higher temperatures (17.5–22.5 °C) are most suitable for corn, which is in agreement with the findings of Agidew (2015); Alemayehu et al. (2020), and Debesa et al. (2020).

Overall, there is a strong connection between land suitability and LULC. Everest et al. (2021) asserted that land suitability analysis is one of the most important methods for sustainable land use. Furthermore, land must be used appropriately to maximize its potential and capabilities (Everest et al., 2021). Owing to limited data availability, only a limited number of factors were considered for the requirements of teff, rice, and corn crops. However, other factors, such as socio-economic and environmental variables, can also be considered when assessing land suitability. Although the technique has been applied only for major crops in the area (teff, rice, and corn), it is also applicable for other crops (e.g., pulses and oilseeds).

## Conclusions

On the basis of the LULC analysis, a pronounced increase in agricultural area in all three catchments during the last three decades was found. This is due to population growth and the associated increased demand for land and wood, e.g., resulting in the clearing of forests for fuel and construction purposes and as a source of income. Deforestation also causes soil erosion and nutrient depletion and may decrease land productivity. The major limiting factor for the increase in agriculture is suitable land for conversion. The high percentages of agricultural areas show that the potential for agricultural expansion will be rather limited in the future. Therefore, to meet future demands for food, an intensification of agricultural land is necessary. Past trends in LULC should shift toward better resource management, e.g., conservation of existing vegetation, afforestation to conserve natural resources, and maintaining the productivity of the soil by creating awareness for appropriate land management practices.

Land suitability for agriculture is significant in agricultural development and future planning. Most parts of the Gilgelabay, Gumara, and Ribb catchments were moderately suitable for the given crops. With respect to future developments, using projected climate data for RCP4.5 and RCP8.5 showed that the proportion of suitable land for corn increased in all catchments. This is primarily associated with an increase in temperature. The increase in suitable areas for rice in the Gumara and Ribb catchments can be related to an increase in rainfall. Based on these findings, crop land suitability will change in the future. Land suitability assessment for agricultural purposes can help decision makers determine how appropriate a specific use of the land is in a specific location for a certain crop. Thus, land suitability is essential for maximizing crop production by using land according to its potential and capabilities. Moreover, the applied methodology can be transferred to other regions and is applicable for other crops (e.g., pulses, oilseeds) as well.

### Recommendation

In summary, the following measures can be recommended to mitigate the impacts of LULC change and potential land suitability evaluation for major crops:

Addressing deforestation:

- Conservation measures through an afforestation program are an immediate requirement. In addition, planted trees should be protected.
- Alternatives to using timber for fuel must be given to inhabitants to decrease deforestation and protect the environment.
- Income alternatives should be created so that local inhabitants do not need to resort to selling forest products.

Addressing agricultural expansion:

- Provide fertilizers at low costs to intensify agriculture instead of converting other areas to new agricultural land.
- Farming practices can be upgraded by using the Integrated Decision Support System (IDSS) to solve complex issues related to agricultural systems with the aid of concerned stakeholders.

Addressing the drivers of LULC change:

- Create awareness of family planning to manage population growth by relevant experts.
- Raising awareness throughout the region about the optimal use of natural resources, conservation systems, and their benefits. This could play a significant role in the rehabilitation of the environment.

Monitoring LULC change:

LULC changes in the catchments should be monitored on a regular basis to assess whether the observed trends persist.

## Data Availability

The data for this research is available from the first author upon request.

## Appendix

### Accuracy assessments

#### Gilgelabay confusion matrix

**Table 13:** Table A1: Confusion matrix for validation of the 1988 land use and land cover map

	Classified types determined from reference source								
Class types determined from classified map	Plots	Agriculture	Forest	Grass land	Shrub land	Water	Total	User accuracy (%)	F1 score
	Agriculture	103	3	6	3	1	116	88.79	0.92
	Forest	3	52	2	0	0	57	91.22	0.92
	Grassland	2	1	35	1	1	40	87.50	0.81
	Shrubland	1	0	2	22	0	25	88.00	0.86
	Water	0	0	1	0	21	22	95.45	0.93
	Total	109	56	46	26	23	260		
	Producer accuracy (%)	94.49	92.85	76.09	84.62	91.30			

**Table 14:** Table A2: Confusion matrix for validation of the 1998 land use and land cover map

	Classified types determined from reference source								
Class types determined from classified map	Plots	Agriculture	Forest	Grass land	Shrub land	Water	Total	User accuracy (%)	F1 score
	Agriculture	94	5	3	3	0	105	89.52	0.9
	Forest	3	63	3	0	0	69	91.30	0.91
	Grassland	3	0	21	1	0	25	84.00	0.8
	Shrubland	3	1	1	36	0	41	87.80	0.89
	Water	0	0	0	0	20	20	100	1
	Total	103	69	28	40	20	260		
	Producer accuracy (%)	91.26	91.30	75.00	90.00	100			

**Table 15:** Table A3: Confusion matrix for validation of the 2017 land use and land cover map

	Classified types determined from reference source								
Class types determined from classified map	Plots	Agriculture	Forest	Grass land	Shrub land	Water	Total	User accuracy (%)	F1 score
	Agriculture	110	2	4	4	0	120	91.67	0.92
	Forest	1	31	2	1	0	35	88.57	0.89
	Grassland	3	2	26	0	0	31	83.87	0.81
	Shrubland	5	0	1	42	0	48	87.50	0.88
	Water	0	0	0	0	26	26	100	1
	Total	119	35	33	47	26	260		
	Producer accuracy (%)	92.44	88.57	78.79	89.36	100			

### Gumara confusion matrix

**Table 16:** Table A4: Confusion matrix for validation of the 1988 land use and land cover map

	Classified types determined from reference source								
Class types determined from classified map	Plots	Agriculture	Forest	Grass land	Shrub land	Water	Total	User accuracy (%)	F1 score
	Agriculture	104	4	3	4	0	115	90.43	0.92
	Forest	2	32	1	2	0	37	86.49	0.86
	Grassland	3	1	26	0	0	30	86.67	0.85
	Shrubland	3	0	1	56	0	60	93.33	0.92
	Water	0	0	0	0	8	8	100	1
	Total	112	37	31	62	8	250		
	Producer accuracy (%)	92.86	86.49	83.87	90.32	100			

**Table 17:** Table A5: Confusion matrix for validation of the 1998 land use and land cover map

	Classified types determined from reference source								
Class types determined from classified map	Plots	Agriculture	Forest	Grass land	Shrub land	Water	Totals	User accuracy (%)	F1 score
	Agriculture	118	2	3	4	0	127	92.91	0.94
	Forest	2	29	0	0	0	31	93.55	0.89
	Grassland	2	0	28	0	0	30	93.33	0.89
	Shrubland	3	3	2	47	0	55	85.45	0.89
	Water	0	0	0	0	7	7	100	1
	Total	125	34	33	51	7	250		
	Producer accuracy (%)	94.40	85.29	84.85	92.16	100			

**Table 18:** Table A6: Confusion matrix for validation of the 2017 land use and land cover map

	Classified types determined from reference source								
Class types determined from classified map	Plots	Agriculture	Forest	Grassland	Shrub land	Water	Total	User accuracy (%)	F1 score
	Agriculture	117	3	3	4	0	127	92.13	0.93
	Forest	2	29	0	0	0	31	93.55	0.86
	Grassland	2	0	28	0	0	30	93.33	0.89
	Shrubland	3	4	2	46	0	55	83.64	0.88
	Water	0	0	0	0	7	7	100	1
	Total	124	36	33	50	7	250		
	Producer accuracy (%)	94.35	80.56	84.85	92	100			

### Ribb confusion matrix

**Table 19:** Table A7: Confusion matrix for validation of the 1988 land use and land cover map

	Classified types determined from reference source								
Class types determined from classified map	Plots	Agriculture	Forest	Grass land	Shrub land	Water	Total	User accuracy (%)	F1 score
	Agriculture	104	2	4	4	0	114	91.23	0.92
	Forest	2	19	1	1	0	23	82.62	0.84
	Grassland	4	0	49	1	0	54	90.74	0.9
	Shrubland	3	1	1	47	0	52	90.38	0.89
	Water	0	0	0	0	7	7	100	1
	Total	113	22	55	53	7	250		
	Producer accuracy (%)	92.04	86.36	89.09	88.68	100			

**Table 20:** Table A8: Confusion matrix for validation of the 1998 land use and land cover map

	Classified types determined from reference source								
Class types determined from classified map	Plots	Agriculture	Forest	Grassland	Shrubland	Water	Total	User accuracy (%)	F1 score
	Agriculture	109	3	4	2	0	118	92.37	0.93
	Forest	1	25	2	1	0	29	86.21	0.85
	Grassland	4	1	52	1	0	58	89.66	0.89
	Shrubland	2	1	0	36	0	39	92.31	0.91
	Water	0	0	0	0	6	6	100	1
	Total	116	30	58	40	6	250		
	Producer accuracy (%)	93.96	83.33	89.66	90	100			

**Table 21:** Table A9: Confusion matrix for validation of the 2017 land use and land cover map

	Classified types determined from reference source								
Class types determined from classified map	Plots	Agriculture	Forest	Grass land	Shrub land	Water	Total	User accuracy (%)	F1 score
	Agriculture	106	3	5	2	0	116	91.38	0.92
	Forest	2	38	1	1	0	42	90.48	0.89
	Grassland	4	1	39	1	0	45	86.67	0.86
	Shrubland	2	1	1	30	0	34	88.24	0.88
	Water	0	0	0	0	13	13	100	1
	Total	114	43	46	34	13	250		
	Producer accuracy (%)	92.98	88.37	84.78	88.24	100			

**Table 22:** Table A10: Land use requirements for rainfed agriculture based on land characteristics (FAO, 1984; Dent & Ridgway, 1986; Seyfu, 1997; Mohammed, 2003; Lupia, 2012; Asmaamw et al., 2015; Ayehu&Besufekad, 2015; Esa&Assen, 2017)

Crop types						
**Rice**						
Land quality and code	Diagnostic factors	Unit	S1	S2	S3	N
Moisture availability (m)	Mean rainfall	mm	> 1400	900–1400	500–900	< 500
Temperature regime (T)	Temperature	°C	20–30	18–20	> 30	< 18
	Altitude	m	0–600	600–1200	1200–1800	> 1800
Land workability (p)	Texture,	Class	C, SC, SCL	S,CL	SL,L	Sandy
Erosion hazard (e)	Slope gradient	%	0–4	4–8	8–20	> 20
**Corn**						
Moisture availability (m)	Mean rainfall	mm	500–750	400–500;750–1200	300–400;1200–1600	< 300; > 1600
Temperature regime (T)	Temperature	Co	17.5–22.5	15–17.5; 22.5–25	13–15; 25–32	< 13; > 32
	Altitude	m	1500–2200	1000–1500;2200–2400	2400–3200	< 1000; > 3200
Land workability (p)	Texture,	Class1	S, LS, SL, L	SiL, Si, SCL,CL, SiCL	SC, SiC, C	SC, SiC, C
Erosion hazard (e)	Slope gradient	%	0–5	0–15	15–30	> 30
**Teff**						
Moisture availability (m)	Mean rainfall	mm	400–550	300–400; 550–800	200–300;800–1200	< 200; > 1200
Temperature regime (T)	Temperature	Co	15–20	12.5–15; 20–25	25–30	< 12.5; > 30
	Altitude	m	1800–2200	1000–1600;1600–1800;2200–2400	2400–2800	< 1000; > 2800
Land workability (p)	Texture,	Class1	S, LS, SL,L	SiL, Si, SCL,CL, SiCL	SC, SiC, C	HC
Erosion hazard (e)	Slope gradient	%	0–5	0–15	15–30	> 30

**Table 23:** Table A11: Land suitability area proportion in ha and percentage of major crops of the Gilgelabay, Gumara and Ribb catchments

Gilgelabay	Baseline	Teff	%	Corn	%		
	N	51,430.6	17.9	15,629.1	5.4		
	S1	95,003.4	33.1	96,057.6	33.4		
	S2	126,318.1	43.9	118,001.3	41.1		
	S3	14,681.5	5.1	57,745.5	20.1		
		287,433.5	100.0	287,433.5	100.0		
	RCP4.5	Teff	%	Corn	%		
	N	51,430.6	17.9	15,629.1	5.4		
	S1	75,552.6	26.3	109,260.6	38.0		
	S2	88,223.4	30.7	119,921.2	41.7		
	S3	72,227.0	25.1	42,622.6	14.8		
		287,433.5	100.0	287,433.5	100.0		
	RCP8.5	Teff	%	Corn	%		
	N	51,430.6	17.9	15,629.1	5.4		
	S1	75,552.6	26.3	109,260.6	38.0		
	S2	88,223.4	30.7	119,921.2	41.7		
	S3	72,227.0	25.1	42,622.6	14.8		
		287,433.5	100.0	287,433.5	100.0		
Gumara	Baseline	Teff	%	Corn	%	Rice	%
	N	16,434.4	15.1	2468.2	2.3	27,243.7	25.0
	S1	23,327.4	21.4	22,439.3	20.6	18,548.2	17.0
	S2	53,687.5	49.2	31,153.2	28.6	35,044.3	32.1
	S3	15,650.2	14.3	53,038.8	48.6	28,263.3	25.9
		109,099.5	100.0	109,099.5	100.0	109,099.5	100.0
	RCP4.5	Teff	%	Corn	%	Rice	%
	N	16,434.4	15.1	2468.2	2.3	27,243.7	25.0
	S1	23,327.4	21.4	37,990.3	34.8	24,961.7	22.9
	S2	53,687.5	49.2	43,865.5	40.2	28,630.8	26.2
	S3	15,650.2	14.3	24,775.6	22.7	28,263.3	25.9
		109,099.5	100.0	109,099.5	100.0	109,099.5	100.0
	RCP8.5	Teff	%	Corn	%	Rice	%
	N	16,434.4	15.1	2468.2	2.3	27,243.7	25.0
	S1	23,327.4	21.4	37,990.3	34.8	24,961.7	22.9
	S2	53,687.5	49.2	43,865.5	40.2	28,630.8	26.2
	S3	15,650.2	14.3	24,775.6	22.7	28,263.3	25.9
		109,099.5	100.0	109,099.5	100.0	109,099.5	100.0
Ribb	Baseline	Teff	%	Corn	%	Rice	%
	N	6380.2	4.5	2399.6	1.7	15,868.6	11.1
	S1	28,894.7	20.2	60,391.9	42.1	52,407.4	36.6
	S2	94,233.1	65.7	38,977.6	27.2	46,962.1	32.8
	S3	13,827.2	9.6	41,566.0	29.0	28,097.0	19.6
		143,335.1	100.0	143,335.1	100.0	143,335.1	100.0
	RCP4.5	Teff	%	Corn	%	Rice	%
	N	6380.2	4.5	2399.6	1.7	15,868.6	11.1
	S1	28,894.7	20.2	78,610.5	54.8	62,859.2	43.9
	S2	94,233.1	65.7	44,517.2	31.1	36,510.3	25.5
	S3	13,827.2	9.6	17,807.7	12.4	28,097.0	19.6
		143,335.1	100.0	143,335.1	100.0	143,335.1	100.0
	RCP8.5	Teff	%	Corn	%	Rice	%
	N	6380.2	4.5	2399.6	1.7	15,868.6	11.1
	S1	28,894.7	20.2	78,610.5	54.8	62,859.2	43.9
	S2	94,233.1	65.7	44,517.2	31.1	36,510.3	25.5
	S3	13,827.2	9.6	17,807.7	12.4	28,097.0	19.6
		143,335.1	100.0	143,335.1	100.0	143,335.1	100.0

## Declarations

All authors have read, understood, and complied as applicable with the statement on "Ethical responsibilities of Authors", as found in the Instructions for Authors.
